# RAD51 separation of function mutation disables replication fork maintenance but preserves DSB repair

**DOI:** 10.1016/j.isci.2024.109524

**Published:** 2024-03-16

**Authors:** Mi Young Son, Ondrej Belan, Mario Spirek, Jakub Cibulka, Fedor Nikulenkov, You Young Kim, Sunyoung Hwang, Kyungjae Myung, Cristina Montagna, Tae Moon Kim, Lumir Krejci, Paul Hasty

**Affiliations:** 1 Department of Molecular Medicine, The Barshop Institute for Longevity and Aging Studies, The Cancer Therapy Research Center, UT Health San Antonio, San Antonio, TX 78229, USA; 2 Department of Biology, Masaryk University, 625 00 Brno, Czech Republic; 3 Center for Genomic Integrity Institute for Basic Science (IBS), Ulsan 44919, Republic of Korea; 4 Department of Genetics, Albert Einstein College of Medicine of Yeshiva University, Bronx, NY 10461, USA; 5 National Centre for Biomolecular Research, Masaryk University, 625 00 Brno, Czech Republic

**Keywords:** Properties of biomolecules, Genetics, Molecular biology, Molecular interaction

## Abstract

Homologous recombination (HR) protects replication forks (RFs) and repairs DNA double-strand breaks (DSBs). Within HR, BRCA2 regulates RAD51 via two interaction regions: the BRC repeats to form filaments on single-stranded DNA and exon 27 (Ex27) to stabilize the filament. Here, we identified a RAD51 S181P mutant that selectively disrupted the RAD51-Ex27 association while maintaining interaction with BRC repeat and proficiently forming filaments capable of DNA binding and strand invasion. Interestingly, RAD51 S181P was defective for RF protection/restart but proficient for DSB repair. Our data suggest that Ex27-mediated stabilization of RAD51 filaments is required for the protection of RFs, while it seems dispensable for the repair of DSBs.

## Highlights

• RAD51 SP mutant is selectively deficient in Ex27-binding
• Ex27 failed to protect SP filaments against BRC4 but does not affect ATP hydrolysis
• RAD51 SP is proficient for HR-mediated DSB repair but fails to protect RFs
• Loss of the RAD51-Ex27 interaction results in increased Robertsonian translocations

## Graphical abstract

**Figure:**
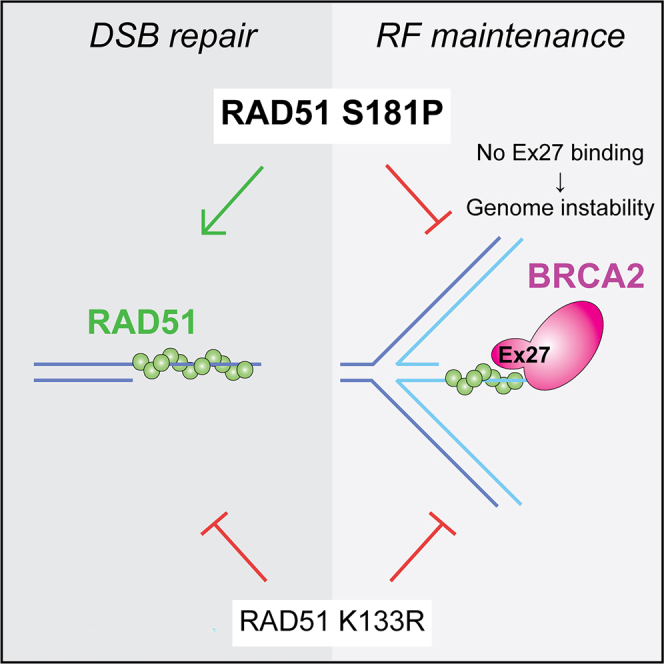

## Introduction

Homologous recombination (HR) repairs DNA double-strand breaks (DSBs), restarts stalled replication forks (RFs) and protects them from nucleolytic degradation.^1^^,^^2^^,^^3^^,^^4^^,^^5^^,^^6^^,^^7^^,^^8^ BRCA2 is a recombination mediator suppressing hereditary breast and ovarian cancer.^9^ To mediate HR, BRCA2 associates with RAD51 via two regions: the BRC repeats^10^^,^^11^ and exon 27 (Ex27).^12^^,^^13^ RAD51-BRC interaction nucleates RAD51 to DNA damage sites on single-stranded ssDNA and promotes displacement of RPA, formation of helical RAD51-ssDNA nucleoprotein filament and strand exchange.^14^^,^^15^^,^^16^^,^^17^^,^^18^ Mechanistically, BRC4 or BRC 1–8 stimulates RAD51 binding to ssDNA^19^ and BRC repeats (BRC1-4) block RAD51 ATP hydrolysis permitting the accumulation of an ATP-bound nucleoprotein filament.^20^ Ex27 binds to an interface created by two adjacent RAD51 monomers^21^^,^^22^ to protect and restart RFs.^23^ Cells expressing BRCA2 deleted for Ex27 exhibit hypersensitivity to γ-radiation, premature replicative senescence, chromosomal instability, increased stalled RF, and reduced survival due to early cancer onset.^2^^,^^24^^,^^25^

Here, we elaborate on the role of the RAD51-Ex27 interaction. We show that Ex27 does not influence ATP hydrolysis but stabilizes RAD51 filaments from dissociation in the presence of a GST-BRC3 trap. We identify a mutation of serine 181 (S181P) that selectively inhibits the interaction between RAD51 and Ex27. In contrast to the well-described RAD51 K133R mutant, RAD51 S181P is able to hydrolyze ATP but Ex27 cannot stabilize the filament. Importantly, the RAD51-Ex27 association is dispensable for repairing DSBs but is required for RAD51-mediated protection of stalled RFs to preserve chromosomal integrity.

## Results

### RAD51 S181P reduces the interaction with Ex27

**Figure 1:**
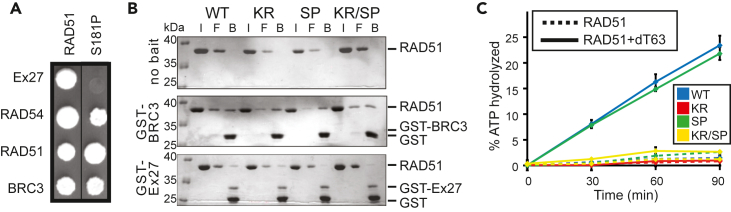
RAD51 S181P (SP) is selectively deficient for the interaction with BRCA2 Ex27 (A) Yeast-two hybrid showing that SP associates with RAD54, RAD51, and BRC3 but not with Ex27. (B) *In vitro* pull-down showing SP interaction with RAD51 and BRC3 but not with Ex27. Gels stained with Coomassie Blue. I = input, F = flow-through, B = bound fractions. (C) The ATPase activity of RAD51 WT, KR, SP, and KR/SP in the absence (dotted lines) or presence of ssDNA [(dT)63] (solid lines).

The mutagenic yeast-two hybrid protocol^26^ was used to isolate a mutation that diminished RAD51’s interaction with Ex27 but maintained interactions with RAD54, BRC3, and itself. Upon screening ∼2,500 colonies, a mutant was isolated that failed to grow with Ex27 but grew with the other partners (Figure 1A) and sequencing revealed a serine to proline change in residue 181 (S181P or SP). RAD51 S181P interaction with BRC3 but not with Ex27 was validated by co-immunoprecipitation (Figure S1A) and further confirmed in an *in vitro* pull-down assay using purified proteins (Figure 1B). To determine the requirement of specific change from serine 181 to proline for interaction with Ex27, we generated and tested recombinant RAD51 S181A and S181R mutants. By using biolayer interferometry (BLI) and *in vitro* pull-down assays, we observed that both S181A and S181R were capable of associating with Ex27 (Figures S1B and S1C), suggesting that loss of interaction can be attributed to the proline residue.

### Ex27 peptide fails to bind and stabilize RAD51 SP filaments *in vitro*

Previous studies have proposed that Ex27-RAD51 interaction is important for protecting stalled RFs against MRE11-mediated degradation but is dispensable for HR-dependent repair of DSBs.^6^ For comparison purposes, we purified and examined DSB repair-deficient RAD51 K133R (KR) mutant that is Ex27-interaction proficient (Figure 1B) but, in contrast to RAD51 SP, deficient in ATPase activity^27^ (Figure 1C). A double-mutant, RAD51 K133R/S181P (KR/SP) was also generated, which displayed deficiency in both Ex27-interaction and ATPase activity (Figures 1B and 1C).

**Figure 2:**
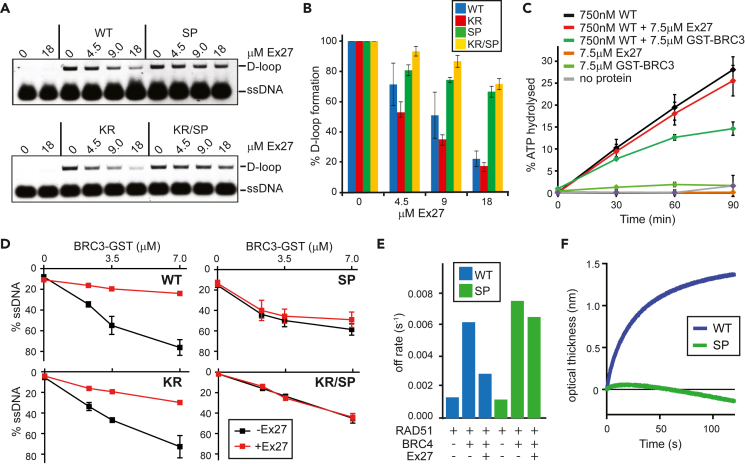
RAD51 SP filaments were proficient for D-loop formation, but not Ex27 stabilization (A) Increasing amount of Ex27 inhibited D-loops for WT and KR, but not SP and KR/SP. Assay was performed in the presence of calcium and ATP to obtain similar levels of basal D-loop between all mutants. (B) Quantification of gels shown in panel A (Mean ± SD, n = 3). (C) Unlike GST-BRC3 (7.5 μM), Ex27 failed to inhibit the ATPase activity of WT (Mean ± SD, n = 3). (D) Ex27 peptide (7.5 μM) failed to stabilize SP and KR/SP protein-DNA complexes from BRC3-GST mediated disassembly (Mean ± SD, n = 3). (E) Ex27 failed to protect SP filaments against dissociation by BRC4 peptide monitored by BLI. WT (blue) and SP (green) filaments were pre-formed on immobilized ssDNA (oligo-dT, 43-mer), and RAD51 dissociation was followed in the presence of BRC4 and Ex27. Off-rates of RAD51 dissociations are plotted, for dissociation curves in Figures S2F and S2G. (F) Binding of Ex27 to RAD51-ssDNA filaments using BLI. RAD51 WT (blue) and SP (green) filaments were formed as aforementioned and the binding of Ex27 was detected in real time as an increase in optical thickness.

Next, we analyzed the biochemical properties of SP both individually and in combination with Ex27 and compared them to KR and KR/SP. Using a D-loop assay in the presence of magnesium ions, we show that RAD51 wildtype (WT) and SP displayed comparable levels of D-loops (Figure S2A). Conversely, KR and KR/SP mutants showed significantly higher D-loop yields under the same conditions (Figure S2A), which correlates with deficient ATPase activity and formation of more stable ATP-bound filaments. In the presence of calcium ion, which induced comparable levels of D-loop for all tested proteins, the addition of Ex27 hindered D-loop formation for WT and KR, while SP and KR/SP mutants remained resistant to this inhibition (Figures 2A and 2B). BRC repeats were shown to inhibit RAD51 ATPase activity;^14^ therefore, we tested the influenced of ATPase activity on RAD51-ssDNA complexes. Unlike GST-BRC3, Ex27 peptide did not affect RAD51 ATPase activity, even at a 10-fold excess over RAD51 WT (Figure 2C).

To examine whether RAD51 SP filaments fail to be protected against BRC destabilization in the presence of Ex27, pre-formed RAD51 filaments on fluorescently labeled ssDNA were exposed to Ex27 peptide and subsequently challenged with GST-BRC3 to monitor RAD51 dissociation in a gel shift assay. The binding of Ex27 to RAD51-ssDNA filaments was evident as an additional super-shift of migrating RAD51-ssDNA complexes for WT and KR but not for SP and KR/SP (Figure S2B). Upon challenge by GST-BRC3, free ssDNA was released from RAD51-ssDNA complexes, indicating complex disassembly (Figure S2B). In the presence of Ex27, RAD51 WT, and KR-ssDNA filaments are almost completely resistant to GST-BRC3, suggesting stabilization by Ex27 (Figure 2D). In contrast, SP and KR/SP filaments were not protected (Figures 2D and S2B), further supporting the loss of Ex27 interaction. These data indicate that while Ex27 cannot inhibit the ATPase activity of RAD51 WT, it does reduce D-loop formation, possibly via binding and stabilization of RAD51-ssDNA filament.

Our previous methods are limited to monitoring protein-DNA binding at the steady state. We thus employed pre-steady state analysis using stopped-flow to address potential alterations in RAD51 filament assembly, specifically focusing on RAD51 SP filaments and the effect of Ex27 (Figures S2C and S2D). This technique allowed us to monitor the kinetics of protein-ssDNA complex assembly in real-time by mixing RAD51 with a 5′-Cy3 fluorescently labeled (dT)_79_ oligonucleotide (Cy3-79-mer).^28^ The binding of RAD51 to 5′-Cy3-(dT)_79_ leads to an increase in Cy3 fluorescence signal due to Cy3 isomerization, known as protein-induced fluorescence enhancement (PIFE). The relative fluorescence change over time serves as a proxy of protein-DNA complex formation. The assembly of RAD51 filaments involves complex binding mechanism,^29^ and we observed a similar mechanism for the SP protein but noted slower kinetics of assembly at suboptimal concentrations (Figure S2E). In the presence of Ex27, we observed changes in the kinetics of WT filament assembly by increasing overall binding amplitude and decreasing binding rate (Figure S2C). In contrast, Ex27 had no effect on the SP mutant (Figure S2D), confirming the loss of interaction.

Furthermore, a BLI-based assay was used to monitor RAD51 filament disassembly.^30^ RAD51 filaments were assembled on biotin-ssDNA immobilized on a streptavidin biosensor tip, and their dissociation by BRC4 peptide was observed over time. The data from the dissociation phase were normalized to the starting point, and the amplitude of the change was plotted as a function of time. While the BRC4 peptide strongly induced the disassembly of both WT and SP filaments, the addition of Ex27 only suppressed RAD51 dissociation from DNA for the WT protein (Figures 2E, S2F, and S2G). Accordingly, no association of Ex27 with SP filament was detected by BLI (Figure 2F).

### RAD51 S181P cells are proficient in HR-mediated DSB repair

To assess the cellular phenotype of human RAD51 WT and SP, a knockout-knockin protocol in mouse embryonic stem (ES) cells (AB2.2) was used to introduce corresponding cDNAs adjacent to the mouse *RAD51* promoter while leaving the remaining mouse allele intact.^31^ *RAD51* KR and KR/SP cDNAs were also included. An essential component of this protocol is the *miniHPRT* gene, which can be selected for or against expression using HAT (hypoxanthine, aminopterin, thymidine) and 6-thioguanine (TG), respectively^32^^,^^33^ (Figure S3A). The genotypes are described as a mouse: AB2.2 RAD51^WT/+^ or AB2.2 RAD51^(KR, SP, KR/SP)/+^ and will be referred to as WT, KR, SP, and KR/SP for this paper. In contrast to previously reported reduced clonogenicity of KR mutant,^2^ the SP had no effect compared to WT (data not shown).

**Figure 3:**
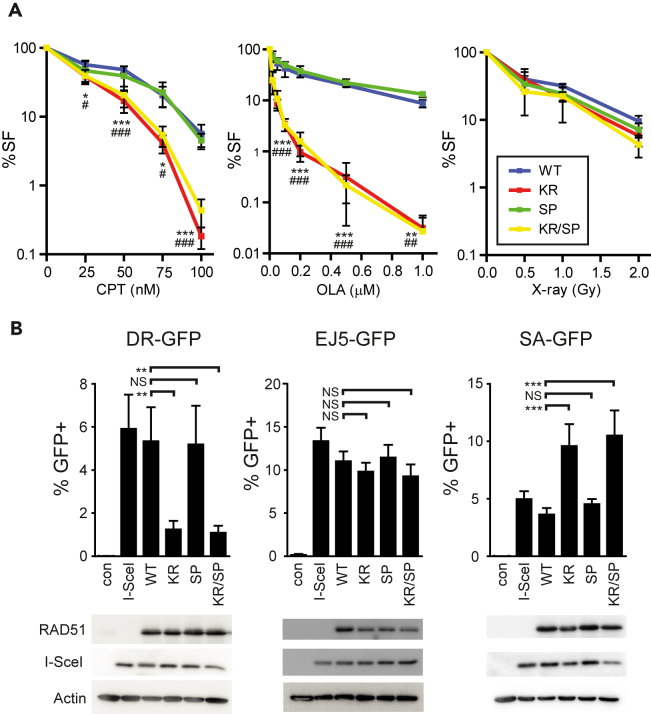
RAD51 interaction with BRCA2 Ex27 is dispensable for HR-mediated DSB repair (A) Dose response of cells expressing RAD51 WT, KR, SP, and KR/SP to CPT, OLA, and X-rays. Percentage of survival fraction (SF) is shown as a mean ± SD (n = 3). Statistics: two-way ANOVA with Tukey’s multiple comparisons test. WT vs. KR: ∗ <0.05, ∗∗ <0.005, ∗∗∗ <0.0005. For WT vs. KR/SP: # <0.05, ## <0.005, ### <0.0005. (B) HR (DR-GFP), NHEJ (EJ5-GFP), SSA (SA-GFP) assays in U2OS cells. Percentage of GFP positive cell is shown as a mean ± SD (n = 3, 4 in case of DR-GFP). Statistics: One-way ANOVA with Tukey’s multiple Comparison Test. Not significant (NS), ∗ <0.05, ∗∗ <0.01, ∗∗∗ <0.001. Levels of RAD51, HA-tagged I-Sce1, and Actin (loading control) for these experiments are presented at the bottom.

A dose-response curve was recorded for camptothecin (CPT, type 1 topoisomerase inhibitor), olaparib (OLA, PARP1 inhibitor), and X-rays to assess the response to clastogenic agents. CPT and OLA induce damage associated with replication,^34^^,^^35^ while X-rays cause mainly replication-independent DSBs plus a variety of replication-associated damage, including modified bases, single-strand breaks, and protein-DNA crosslinks.^36^ Treatment with CPT and OLA was initiated upon the cell-seeding and continued throughout the experiment.^37^ Cell number was counted five days later. Unlike KR and KR/SP cells, SP cells were not hypersensitive to CPT and OLA (Figure 3A, left & middle panels); however, none of these mutants displayed differential sensitization to X-rays (Figure 3A, right panel), possibly reflecting replication requirement for CPT and OLA-induced damage.

The proficiency of DSB repair via HR was evaluated using the DR-GFP assay in U2OS cells overexpressing RAD51 variants. KR and KR/SP cells showed a significant drop in number of GFP-positive cells (Figure 3B, left panel), consistently with a previously reported dominant-negative effect of KR expression on HR efficiency.^27^ In contrast, SP cells exhibited a similar number of GFP-positive cells compared to WT, indicating their comparable HR proficiency. To assess the efficiency of other DSB repair pathways, the EJ5-GFP and SA-GFP assays were performed in U2OS cells. The EJ5-GFP assay measures non-homologous end-joining (NHEJ), a pathway that rejoins DNA ends without using a homologous template. In contrast, the SA-GFP assay evaluated single-strand annealing (SSA), which joins complementary ends when repeats flank the DSB.^38^^,^^39^ The NHEJ levels were comparable in all cell lines (Figure 3B, middle panel) while the level of SSA was significantly elevated in KR and KR/SP cells, pointing to a compensatory mechanism in these HR-deficient cells (Figure 3B, right panel). These observations cannot be explained by differential RAD51 expression levels as shown by western blot (Figure 3B, bottom). Our data suggest that the RAD51-Ex27 interaction is dispensable for DSB repair via HR, while RAD51 ATPase activity is critical. Accordingly, WT and SP cells were proficient in ATPase activity, while KR and KR/SP were not (Figure 1C).

### RAD51 SP forms foci in cells but fails to alleviate replication stress

**Figure 4:**
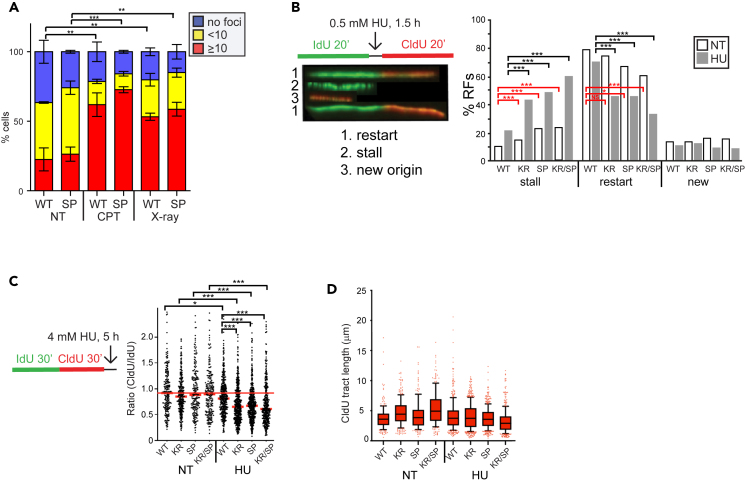
Analysis of replication fork dynamics in RAD51 mutant-expression cells (A) Graph summarizing the percentages of RAD51 foci for cells expressing eGFP-WT or eGFP-SP, either untreated (NT) or exposed to 1 μM CPT for 3 h or 1 Gy X-rays, followed by 1 h recovery. More than 184 cells were counted per each sample and the counting assessment was done blind. The graph is showing the percentage of cells without (no) or with foci (<10 or ≥ 10) as a mean ± SD (n = 3). Statistics (unpaired t-test) is indicated for the ≥ 10 foci dataset: ∗∗p < 0.005, ∗∗∗p < 0.0005. (B) Analysis for RF progression by DNA fiber assay. Left panel: Schematics of the assay, cells were exposed to IdU for 20 min, then a mild HU dose (0.5 mM, 1.5 h), followed by drug removal and treatment with CldU for 20 min. Three categories of phenotypes were scored: 1) restarted RF (green-red), 2) stalled RF (green), 3) new origin (red). At least 1000 fibers were analyzed for each sample. Statistics: a Chi-square with Yates’ correction and Fisher’s exact test. (C) Analysis for nascent strand degradation. Schematics of the assay: cells were treated with IdU (30 min) followed with CldU (30 min) and then HU (4 mM, 5 h). At least 129 fibers were observed for each sample. Short red lines demarcate the median for each sample, the long red line demarcates the median for WT no treatment (NT). Statistics: Kruskal-Wallis test with Dunn’s multiple comparison. Not significant (NS), ∗p < 0.05, ∗∗p < 0.005, ∗∗∗p < 0.0005. (D) Track length taken from the experiment described in F and converted into kb using conversion factor 1 μm = 2.59 kb.^40^

RAD51 WT and SP foci were visualized with green fluorescence by expressing *RAD51* cDNA conjugated to enhanced green fluorescent protein (eGFP). WT mouse *RAD51* was deleted in cells with eGFP-WT or eGFP-SP (Figure S3A) by using CRISPR/Cas9 (Figure S3B). In this manuscript, we refer to the genotypes as eGFP-WT or eGFP-SP for simplicity. The number of cells with ≥10 foci was comparable between unexposed eGFP-WT and eGFP-SP cells (Figures 4A and S4). Upon exposure to CPT (1 μ M, 3 h) or X-rays (1 Gy, 3 h release), the number of cells with ≥10 foci increased similarly for eGFP-WT and eGFP-SP (Figures 4A and S4).

To test the role of RAD51 mutants in maintaining RFs, we performed dose-response curves to three agents that influence replication: hydroxyurea (HU, a ribonucleotide reductase inhibitor), aphidicolin (APH, an inhibitor of DNA polymerase α, δ, and ε) and VE-821 (an ATR inhibitor). No significant differences were observed in response to these agents (Figures S5A‒S5C). These data are consistent with the mild sensitivity of RAD51 knock-outs to HU and ATRi, as compared to olaparib, reported in pooled CRISPR screens.^41^ Similarly, BRCA2 loss results only in subtle sensitization to HU compared to strong sensitization to olaparib.^6^ Therefore, even if some of the RAD51 mutants confer slight sensitivity to replication poisons in our system, where the RAD51 WT allele is present, these more-subtle effects may not be detectable in cell survival assays. However, HR is known to process stalled RFs after exposure to a mild dose of HU that does not cause breaks.^42^ To quantify stalled and restarted RFs by DNA fiber assay, cells were treated with IdU for 20 min followed by exposure to 0.5 mM HU for 1.5 h, and then CldU was added for 20 min. Compared to WT, all RAD51 mutants exhibited increased RF stalling without HU treatment and after HU treatment. Compared to WT, HU treatment increased RF stalls for all mutants (Figure 4B). In addition, all mutants also showed decrease in fork restart compared to WT (Figure 4B), indicating possible defect in fork protection and/or reversal. Stalled RFs are known to be susceptible to nucleolytic degradation by MRE11 nuclease in the absence of stable RAD51-ssDNA interaction.^6^ To investigate nascent strand protection, the CldU/IdU ratio was measured in cells treated with IdU for 30 min, followed by CldU for 30 min, and then 4 mM HU for 5 h. Compared to WT, the RAD51 mutants showed insignificant degradation without treatment (NT). Yet, all mutants exhibited nascent strand degradation after HU exposure compared to WT (Figure 4C). RF speed was evaluated by observing the length of the CldU fibers. Without HU treatment, CldU tract length showed no difference between WT an SP, yet longer fibers were seen with KR and KR/SP suggesting faster replication (Figure 4D). With HU treatment, the length of the CldU fibers were the same for WT, KR, and SP; however, the KR/SP were shorter suggesting reduced speed (Figure 4D), possibly due to a combination of deficient fork reversal and subsequent fork degradation (Figure 4C).^43^

### RAD51 SP causes a different array of chromosomal aberrations than KR

**Figure 5:**
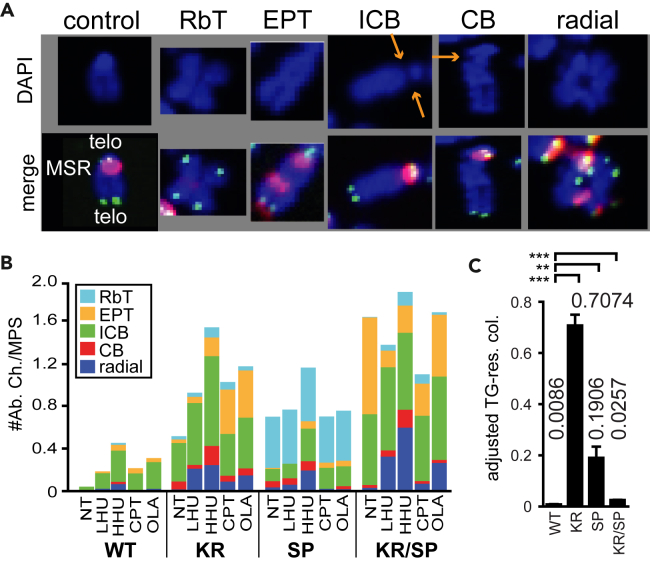
Loss of RAD51-Ex27 interaction resulted in distinct chromosomal abnormalities (A) The representative images of a chromosomes visualized by two-color FISH analysis. The top picture is stained blue with DAPI (4′,6-diamidino-2-phenylindole). The bottom picture is a merge of the pericentromere shown by a red major satellite repeat (MSR) probe and telomeres (telo) shown by a green telomere probe. Different types of chromosomal abnormalities: Robertsonian translocations (RbT), extra pericentromeres and telomeres (EPTs), isochromatid breaks (ICBs), chromatid breaks (CBs), and radials. Arrows point to breaks. (B) Number of abnormal chromosomes (#Ab. Ch) per metaphase spread (MPS). Non-treated (NT), low HU (LHU, 0.5 mM, 1.5 h), high HU (HHU, 4 mM, 5 h), CPT (100 nM), and OLA (1 μM). After treatment, cells were released for 24 h, and then MPS were prepared. At least 97 MPHs were observed for each group. For statistics see Table S1. (C) The *miniHPRT* loss-of-function assay for the RAD51 mutants. Thioguanine-resistant colonies (TG-res. col.). Each number over the bar represents mean ± SD (n = 3). Statistics: Unpaired t-test (two-tailed). ∗p < 0.05, ∗∗p < 0.005, ∗∗∗p < 0.0005.

To investigate the potential differences in chromosomal alterations resulting from the differential requirements of ATPase activity and Ex27 interaction, we assessed chromosome stability using two-color fluorescence *in situ* hybridization (FISH) on metaphase spreads (MPSs).^44^ We scored various chromosomal aberrations, including Robertsonian translocations (RbT), extra pericentromeres and telomeres (EPTs), isochromatid breaks (ICBs), chromatid breaks (CBs), and radials (Figure 5A).^2^ RbTs occur when two chromosomes join at the centromere, with or without telomeres, and could arise due to defective replication or chromosome fusion.^45^ EPTs are duplications resulting from faulty replication or imprecise joining of multiple DSBs and have been described in cells expressing RAD51 K133A,^2^ a mutation that inhibits ATP binding. ICBs suggest failed strand exchange intermediates that break both chromatids, while CBs represent one-ended breaks at collapsed RFs. Radials are chromosomal structures formed by the fusion of multiple chromosomes and occur in cells derived from Fanconi anemia patients exposed to crosslinking agents.^46^ KR, SP, and KR/SP exhibited a 17-, 24-, and 56-fold increase, respectively, in spontaneous chromosomal abnormalities when compared to WT (Figure 5B; Table S1). KR and KR/SP displayed mostly ICBs and ICB/EPT, respectively, consistent with defective DSB repair (Figure 5B; Table S1). SP predominantly showed RbTs without central telomeres, indicating defective replication (Figure 5B; Table S1). Chromosome painting was performed on SP MPSs to further investigate SP cells, revealing two RbTs involving duplication of chromosome 8 with chromosome 8 disomy (Figure S6A).

The effect of dose-dependent genotoxic lesions on chromosomal instability was also analyzed. Cells were exposed to physiologically comparable doses of genotoxins (HU, CPT, and OLA) to ensure that differences in chromosomal instability between genotypes were not related to the dose of the genotoxin (Figures S6B‒S6D). After exposure to genotoxins, KR and KR/SP cells exhibited increased levels of chromosomal defects, notably radials (Figure 5B; Table S1), which could result from elevated levels of NHEJ or SSA (Figure 2D). However, SP cells did not show a significant increase in radials except at a high dose of HU (Figure 5B; Table S1). Furthermore, a *miniHPRT* loss-of-function (LOF) assay was performed to assess the mutation rate in RAD51 variants by measuring cell survival in TG.^31^ KR, SP, and KR/SP exhibited a spontaneous increase of 82-, 22-, and 3-fold, respectively, in TG-resistant colonies compared to WT (Figure 5C). Interestingly, the KR/SP mutant displays a lower mutation level than either KR or SP, suggesting that serine 181 promotes mutagenesis in KR cells, while lysine 133 likely plays a similar, but less prominent role in SP cells. These data support separate function for KR and SP mutants and may reflect use of alternative pathways for repair/mutagenesis. The overall decrease of mutagenesis in the double mutant may be a result of combined loss of both alternative pathways, where most of the damage remains unrepaired and cells mostly die.

## Discussion

Using the yeast two-hybrid screen, we isolated the S181P mutation in RAD51 that is defective in its interaction with BRCA2 exon 27 while retaining its ability to interact with the BRCA2 BRC3 motif and other RAD51-interacting proteins. During the revision process of this manuscript, a cryo-electron microscopy structure of BRCA2 exon27 with RAD51 was published.^47^ This study revealed an acidic patch, containing the RAD51 S181, mediating the interaction with both Ex27 and BRC4. Interestingly, while mutations of the acidic residues (D184, D187) attenuated binding of both Ex27 and BRC4, RAD51 SP mutant is selectively deficient for the interaction with Ex27. The introduction of proline residue is likely responsible for the loss of interaction since exchange of S181 for alanine, arginine, or cysteine did not compromise Ex27 binding (Figure S1;^47^).

Biochemical characterization revealed that the SP mutant maintained ATPase activity, in contrast to KR and KR/SP. Interestingly, Ex27 did not affect the ATPase activity of RAD51-ssDNA complexes but exerted an inhibitory effect on D-loop formation. Previously, Ex27 was shown to stabilize RAD51-ssDNA filaments against BRC3-mediated disassembly.^21^^,^^22^ GST-BRC3 acts as a “RAD51 trap,” binding to dissociated RAD51 monomers and preventing their re-binding to ssDNA.^20^ Our findings show that Ex27 stabilizes RAD51 WT- and KR-ssDNA filaments against dissociation in the presence of GST-BRC3, yet it inhibits their ability to form D-loops. In contrast, SP and KR/SP mutants are dissociated by BRC3 but are not inhibited in forming D-loops, suggesting role of RAD51 turnover during this process. Consistent with these observations, our cellular data indicate that the interaction between Ex27 and RAD51 has a DSB repair-independent function, since SP, unlike KR, did not display a deficiency in HR-mediated DSB repair.

### RAD51 interaction with Ex27 is required for RF maintenance

The RAD51 S181P mutation disabled RF restart and protection against nuclease-mediated degradation. This observation is in accordance with previous studies highlighting the requirement of both BRCA2 BRC repeats and Ex27 for RF maintenance.^6^^,^^7^^,^^48^ A recent study has also shown that in contrast to fork protection, its reversal depends on RAD51’s ability to catalyze strand invasion.^43^ This points to a more specific role of Ex27-dependent regulation of RAD51 filament in controlling DNA accessibility during the processing of stalled forks. Notably, the RAD51 SP mutant differs from the human RAD51-II3A mutant that protects against MRE11 but not against DNA2.^49^ This phenotype is reminiscent of RAD51 T131P mutant, which forms less-stable filaments *in vitro* and results in the accumulation of pRPA on RFs but does not exhibit visible HR deficiency in the DR-GFP assay in cells.^50^^,^^51^

### Deficiency in RAD51 Ex27-interaction or its ATPase activity results in different chromosomal abnormality spectrum

Compared to WT, all RAD51 mutant cells exhibited increased levels of spontaneous chromosomal abnormalities. KR cells predominantly exhibited isochromatid breaks while KR/SP cells showed a combination of isochromatid breaks and ETPs. These data are consistent with KR and KR/SP being defective in both HR-mediated DSB repair and RF maintenance. On the other hand, the most common aberrations observed in SP cells were RbTs. These RbTs did not have central telomers, and chromosome painting revealed a duplication of chromosome 8 with chromosome 8 disomy, suggesting a replication defect at the centromere. It has been reported that HR machinery, including RAD51, is recruited to breaks during G_1_ and HR deficiency causes centromeric instability and chromosomal translocations.^52^ We speculate that the centromeric instability observed in SP cells arises as a consequence of RAD51-mediated processing of stalled RFs in peri-centromeres and centromeres. The ability of SP cells to repair breaks via HR and alternative pathways (NHEJ, SSA) may facilitate the specific formation of RbTs. Our data thus describe novel separation of function mutant that might help dissect the multifaceted role of RAD51 in DSB repair and processing of stalled RFs and explain some of disease-associated mutations in RAD51.^53^

### Limitations to the study

This study is limited to the functional characterization of RAD51 KR and SP in mouse ES and U2OS cells, but no organismal data are presented here (a description of the RAD51^SP/SP^ will be submitted in the near future). Another limitation is the lack of analysis of the functional integration of RAD51 with other aspects of RF maintenance. For example, the impact of KR and SP mutations on DNA damage tolerance (DDT) remains unexplored. Previously we showed that RAD51 and BRCA2 were responsible for the fusion of identical inverted repeats, while DDT was responsible for the fusion of mismatched inverted repeats.^54^ Although we have previously shown that RAD51 K133A mutation relies upon TREX2 function to induce nearly all the mutations in ES cells,^31^ the effect of KR and SP mutants on these fusion events remains unknown. Further investigation of these mutants may provide valuable insight into distinguishing between strand exchange and RF protection and shed light on their utilization by alternative pathways.

## STAR★Methods

### Key resources table

**Table:** 

REAGENT or RESOURCE	SOURCE	IDENTIFIER
**Antibodies**		

Monoclonal Rad51 antibody (D4B10)	Cell Signaling	Cat# 8875 RRID: AB_2721109
Monoclonal ***β***-actin antibody (AC-15)	Sigma-Aldrich	Cat# A5441 RRID: AB_476744
Rabbit IgG HRP linked whole ab	GE Healthcare	Cat# NA934; RRID:AB_772206
Mouse IgG HRP linked whole ab	GE Healthcare	Cat# NA931; RRID:AB_772210
Rat monoclonal anti-BrdU [BU1/75 (ICR1)]	AbD Serotec (Bio-Rad)	Cat# MCA2060T, RRID:AB_10015293
Mouse monoclonal anti-BrdU (B44)	BD Biosciences	Cat# 347580, RRID:AB_10015219
Goat anti-rat AlexaFluor555	Thermo Fisher	Cat# A-21434, RRID:AB_141733
Goat anti-mouse AlexaFluor488	Thermo Fisher	Cat# A-11017, RRID:AB_143160
IRDye 680RD goat anti-rabbit	Li-COR Biosciences	Cat# 926-68071; RRID:AB_10956166
IRDye 800CW goat anti-mouse	Li-COR Biosciences	Cat# 926-32210; RRID:AB_621842
FLAG M2	Sigma	Cat# F-3165 RRID:AB_259529
Living Colors A.v. GFP (JL-8)	Takara	Cat# 632380 RRID:AB_10013427
α-tubulin, clone DM1A	Millipore	Cat# 05-829 RRID:AB_310035
β-tubulin	Abcam	Cat# ab-15568 RRID:AB_2210952
Anti-Rad51 antibody, rabbit serum	Bio-Academia	Cat# 70-002; RRID:AB_3075881
Anti-HA tag antibody	Abcam	Cat# ab9110; RRID:AB_307019
Anti-beta Actin antibody	Abcam	Cat# ab184220

**Bacterial and virus strains**		

mutD5 strain GM4708 (E.coli)	Palmer and Marinus^55^	N/A

**Chemicals, peptides, and recombinant proteins**		

Streptactin Sepharose High Performance	GE Healthcare	Cat# 28935599
L-glutamine	Gibco	Cat# 21051-024
Penicillin	Sigma-Aldrich	Cat# P3032
Streptomycin sulfate	Gibco	Cat# 11860-038
β-mercaptoethanol	Sigma-Aldrich	Cat# M3148
Leukemia inhibitory factor mouse	Gemini Bio	Cat# 400-495
Gelatin	Sigma-Aldrich	Cat# G1890
6-Thioguanine	Sigma-Aldrich	Cat# A4882
50x HAT	Sigma-Aldrich	Cat# H0262
50x HT	Sigma-Aldrich	Cat# H0137
Puromycin	Thermo Fisher Scientific-Gibco	Cat# 1113803
Camptothecin	Sigma-Aldrich	Cat# C9911
Olaparib	Selleckchem	Cat# S1060
Hydroxyurea	Sigma-Aldrich	Cat# H8627
Aphidicolin	Sigma-Aldrich	Cat# A0781
VE-821	Selleckchem	Cat# S8007
Dimethyl sulfoxide (DMSO)	Sigma-Aldrich	Cat# D2650
IdU (5-Iodo-2’-deoxyuridine)	Sigma-Aldrich	Cat# I7125
CldU (5’Chloro-2’-deoxyuridine)	Sigma-Aldrich	Cat# C6891
Tris base	Fisher scientific	Cat# BP152-1
EDTA (ethylenediaminetetraacetic acid)	Sigma-Aldrich	Cat# E5134
SDS (sodium dodecyl sulfate)	Fisher scientific	Cat# BP166-500
HCl (hydrochloric acid)	Millipore	Cat# HX0603-75
BSA (bovine serum albumin)	Sigma-Aldrich	Cat# A9418
Paraformaldehyde	Electron Microscopy Sciences	Cat# 15710
Tween 20	Fisher scientific	Cat# BP337-500
Fluoroshield histology mounting medium	Sigma-Aldrich	Cat# F6182
Methanol	Sigma-Aldrich	Cat# 179337
Acetic acid	Millipore	Cat# AX0073-75
EdU (5-ethynyl-2’-deoxyuridine)	Invitrogen	Cat# E10187
Formaldehyde	Sigma-Aldrich	Cat# F8775
Triton X-100	Sigma-Aldrich	Cat# X100
cOmplete mini protease inhibitor cocktail	Roche	Cat# 11836170001
Streptavidin-agarose beads	Novagen	Cat# 69203-3
NaCl (sodium chloride)	Millipore	Cat# SX0420-1
Immobilon western chemiluminescent HRP substrate	Millipore	Cat# WBKLS0500
Colcemid	Santa Cruz Biotechnology	Cat# SC-202550
KCl (potassium chloride)	Fisher scientific	Cat# P217-500
Sodium citrate	EM science	Cat# SC0445-1
Formamide	Fisher Scientific	Cat# BP227-500
Vectashield mounting medium with DAPI	Vector Laboratories	Cat# H-1200
DMEM+GlutaMAX	Thermo Fisher Scientific-Gibco	Cat# 10566-016
3-amino-1,2,4-triazole (3-AT)	Sigma-Aldrich	Cat# A8056-10G
Pulsed Field Certified Agarose	Bio-Rad	Cat# 162-0137
Safe-Pinky DNA Gel Staining Solution	GenDEPOT	Cat# S1000
PhosSTOP	Roche	Cat# 04 906 837 001
Laemmli Reducing Sample Buffer (4x)	GenDEPOT	Cat# L1100-001
Glutathione agarose resin	Thermo Fisher	Cat# 16100

**Critical commercial assays**		

Click-iT Plus reaction cocktail	Thermo Fisher Scientific	Cat# C10639
PiColorLock Gold Colorimetric Assay Kit	Innova Biosciences	Cat# 303-0030

**Experimental models: Cell lines**		

Mouse: AB2.2 RAD51^WT^	Kim et al.^2^	N/A
Mouse: AB2.2 RAD51^K133R^	Kim et al.^2^	N/A
Mouse: AB2.2 RAD51^S181P^	This paper	N/A
Mouse: AB2.2 RAD51^K133R/S181P^	This paper	N/A
Mouse: AB2.2 RAD51^GFP-WT^/-	This paper	N/A
Mouse: AB2.2 RAD51^GFP-S181P^/-	This paper	N/A
Human: HEK 293T/17	ATCC	Cat# CRL-11268
Human: HEK293T	ATCC	Cat# CRL-3216
Human: U-2 OS	ATCC	Cat# HTB-96
Human: U-2 OS DR-GFP	Gunn et al.^56^	N/A
Human: U-2 OS SA-GFP	Gunn et al.^56^	N/A
Human: U-2 OS EJ5-GFP	Gunn et al.^56^	N/A

**Experimental models: Organisms/strains**		

PJ69-4A (yeast)	James et al.^57^	N/A
PJ69-4α (yeast)	James et al.^57^	N/A

**Oligonucleotides**		

HsRAD51^S181P^ SDM CMKIP 5’-GCTGAGAGGTATGGTCTC**CCT**GGCAGTGATG TCCTGG-3’	This paper	N/A
Rad51 5’g-ki sequencing primer (forward) 5’-TCAAAGGTATGTCGGGAAC-3’	Kim et al.^2^	N/A
SV40pA sequencing primer (reverse) 5’-TGATCATAATCAGCCATAC-3’	Kim et al.^2^	N/A
gRNA (forward) for mutating second copy of mouse Rad51exon 4 5’-CACCCAACTGAGTTTCACCAGCGC-3’	This paper	N/A
gRNA (reverse) for mutating second copy of mouse Rad51 exon 4 5’-AAACGCGCTGGTGAAACTCAGTTG-3’	This paper	N/A
5’FITC-labelled 90mer ssDNA [(5’FAM)AAATCAATC TAAAGTATATATGAGTAAACTTGGTCTGACAGTTA CCAATGCTTAATCAGTGAGGCACCTATCTCAGCG ATCTGTCTATTT]	This paper	N/A
BRC3-GST protein trap	This paper	N/A
(dT)63 homopolymer oligonucleotide	This paper	N/A
5'-Cy3 fluorescently labelled (dT)79 homopolymer oligonucleotide	This paper	N/A
3'-biotinylated ssDNA (oligo-dT, 43-mer)	This paper	N/A

**Recombinant DNA**		

pGAD-hRAD51 (human Rad51)	This paper	N/A
pGAD10-rad51	This paper	N/A
pGBKT7-mRad51 (mouse Rad51)	This paper	N/A
pGBKT7-hBRCA2 BRC3 (1414-1502 a.a.)	This paper	N/A
pGBKT7-mBrca2 Ex27 (3161-3266 a.a.)	This paper	N/A
pGBT9-hRAD54 (1-142 a.a.)	This paper	N/A
pGAD-rad51	This paper	N/A
pX330-U6-Chimeric_BB-CBh-hSpCas9	Addgene 42230	RRID:Addgene_42230
pPGKcrepA	Kim et al.^58^	N/A
CMKIP-RAD51cDNA(WT)	Kim et al.^2^	N/A
CMKIP-Rad51cDNA(K133R)	Kim et al.^2^	N/A
CMKIP-Rad51cDNA(S181P)	This paper	N/A
CMKIP-Rad51cDNA(K133R/S181P)	This paper	N/A
Puro expression plasmid (Expuro2, 6E6)	Kim et al., 2011	N/A
pCAGGS empty vector	Stark et al.^27^	N/A
pCßASce expression vector	Stark et al.^27^	N/A
pCMV-hRad51 WT expression vector	This paper	N/A
pCMV-hRad51 S181P mutant expression vector	This paper	N/A
pCMV-hRad51 K133R mutant expression vector	This paper	N/A
pCMV-hRad51 KR-SP mutant expression vector	This paper	N/A
pCDNA5/FRT/TO-Strep II human Rad51-WT	This paper	N/A
pCDNA5/FRT/TO-Strep II human Rad51-S181P	This paper	N/A
pCDNA5/FRT/TO-Strep II human Rad51-K133R	This paper	N/A
pCDNA5/FRT/TO-Strep II human Rad51-KRSP	This paper	N/A
pCMV-3xFlag-Empty Vector (EV)	This paper	N/A
pCMV-3XFlag-hBRCA2 BRC3	This paper	N/A
pCMV-3XFlag-hBRCA2 Ex27	This paper	N/A
human BRCA2 BRC3	This paper	N/A
BRCA2 Ex27	This paper	N/A
EX27-GST	Carreira and Kowalczykowski,^14^	N/A
pGEX-6-P1-BRCA2 exon27 (3189-3418 a.a.)-GST	This paper	N/A
pGEX-6-P1-BRCA2 exon27 (3265-3330 a.a.)-GST	This paper	N/A
pGEX-6-P1-BRC4-GST	Carreira and Kowalczykowski,^14^	N/A
pGEX-6-P1-BRC3-GST	Carreira and Kowalczykowski,^14^	N/A
pET11c-Rad51 (WT)	Spirek et al.^29^	N/A
pET11c-Rad51 S181P	This study	N/A
pET11c-Rad51 K133R	Spirek et al.^29^	N/A
pET11c-Rad51 S181P K133R	This study	N/A
pBluescript SK(-)	Stratagene	GenBank: X52324.1

**Software and algorithms**		

FlowJo	FlowJo LLC	Version#10
Zen 2.3 pro	Zeiss	RRID:SCR_013672
Prism	GraphPad	RRID:SCR_002798
Multi Gauge V3.2	Fujifilm	N/A
ImageJ	Schneider et al.^51^	RRID:SCR_003070
CRISPOR	Concordet et al.^59^	www.crispor.tefor.net
Odyssey Infrared Imaging System Image studio version 3.0 software	Li-COR Biosciences	RRID:SCR_013715

**Other**		

QuickChange II site-directed mutagenesis kit	Agilent-Stratagene	Cat# 200514
CHEF disposable plug mold, 50-well	Bio-Rad	Cat# 170-3713
Gene Pulser Xcell system	Bio-Rad	Cat# 165-2661
CHEF DR III Variable Angle System	Bio-Rad	Cat# 1703-3702
Ex27 Peptide (TR2 region of BRCA2; aa 3265-3330)	Peptide 2.0	Custom synthesis
High Precision Streptavidin (SAX) Biosensors	ForteBio	Cat# 18-5117
ChemiDoc™	Bio-Rad	N/A

### Resource availability

#### Lead contact

Correspondence and requests for materials should be addressed to and will be fulfilled by the Lead Contact, Paul Hasty (hastyhsc@gmail.com).

#### Materials availability

Plasmids and cell lines generated in this study are available without restrictions and will be fulfilled by the lead contact, Paul Hasty (hastyhsc@gmail.com) upon request.

#### Data and code availability

• All data in this paper will be available from the lead contact upon request.
• This study did not generate datasets or code.
• Any additional information required to reanalyze the data reported in this paper is available from the lead contact upon request.

### Experimental model and study participant details

#### Cell lines

Mouse embryonic stem cells (AB2.2 - XY ES cells) were cultured as previously described.^60^ The cells were cultured on 0.1% gelatin-coated cell culture dishes and grown in Dulbecco’s Modified Eagle’s Medium (Hyclone) supplemented with 15% fetal bovine serum (Gemini Bio), 2 mM glutamine, 30 ug/mL penicillin, 50 ug/mL streptomycin, 10^-4^ M β-mercaptoethanol, and 1000 units/mL 10^7^ mouse leukemia inhibitory factor at 37°C in a 5% CO_2_ humidified incubator. Cell lines were maintained and used at low passage numbers (≤10 passages) but have not been authenticated. The human osteosarcoma cell line U-2 OS (ATCC) was grown in DMEM+GlutaMAX (Thermo Fisher Scientific-Gibco) and 10% fetal bovine serum, supplemented with 100 units/mL penicillin-100 μg/mL streptomycin at 37°C, 5% CO_2_ incubator.

The human embryonic kidney cell line HEK 293T/17 (ATCC) was cultured in Dulbecco’s modified Eagle’s medium (DMEM) and 10% fetal bovine serum (Hyclone), supplemented with and 100 units/mL penicillin-100 μg/mL streptomycin at 37°C, 5% CO_2_ incubator.

### Method details

#### Mutagenic yeast-two hybrid screen

Mutations in yeast-two hybrid pGAD-hRAD51 (human RAD51) plasmid were introduced through an Escherichia coli mutD5 mutator strain, GM4708 (PMID: 11154282). Mutagenesis was induced by cultivation for 21 hr in Luria-Bertani (LB) medium containing ampicillin (75 mg/mL). The pool of mutated plasmids (pGAD10-rad51) was transformed into the yeast strain PJ69-4α.^57^ Approximately 6,000 transformants were then manually patched on selective media so each colony would be easily identified by its position, with its row and column numbers in a chess-like pattern. Subsequently, each plate (140 mm) containing individual colonies in PJ69-4α strain was then replica-plated onto four lawns of PJ69-4A strain, each with a different fusion plasmid: pGBKT7-mRad51 (mouse Rad51), pGBKT7-hBRCA2 BRC3 (1414-1502 a.a.), pGBKT7-mBrca2 Ex27 (3161-3226 a.a.: ALDFLSRLPLPPPVSPICTFVSPAAQKAFQPPRSCG)^22^ or pGBT9-hRAD54 (1-142 a.a). After mating, diploids were recovered on SC-Trp-Leu plates and then replica-plated on SC-Trp-Leu-His triple-dropout plates supplemented with 30 mM 3-amino-1,2,4-triazole (3-AT), followed by incubation at 30°C. The pGAD-rad51 plasmids from candidate colonies were isolated and subsequently sequenced to identify the introduced mutations. The organized pattern of colonies facilitated a comparison of their ability to interact with mRad51, BRCA2 BRC3, mBRCA2 Ex27, or hRAD54 proteins, respectively. Consequently, colonies exhibiting disrupted specific interactions were disrupted while others remained intact could be isolated, and then further confirmed to clarify the impact of the mutation on protein-protein interaction.

#### Dose response curves

Done as previously described^37^ with modifications. On Day 0, 2x10^3^ cells were seeded onto 8 inner wells of a 24-well culture plate. On Day 1, cells were treated with CPT (25, 50, 75, 100 nM), OLA (0.02, 0.05, 0.1, 0.2, 0.5, 1 μM), X-ray (0.5, 1, 2 Gy), HU (20, 40, 60, 80 μM), APH (100, 150, 200, 250 nM) and VE-821 (0.4, 0.8, 1.2, 1.6 μM). CPT, OLA, APH and VE-821 were dissolved in DMSO and HU was dissolved in water. On Day 6, the cells were harvested and counted using a hemacytometer. The experiment was performed three times.

#### Targeting *RAD51*

The knockout-knockin protocol has been described for *RAD51*.^2^^,^^58^ Briefly, 5 μg of *MmRAD51* targeting vector containing *SAβgeo* and *miniHPRT* (linearized with PacI) was electroporated to the cells and they were selected with 1x HAT and 2x G418. Targeted cells were transfected with 10 μg of pPGKcrepA to remove 5’ half of *miniHPRT* cassette and selected in 1x 6-TG. Then, 20 μg of Cre-mediated targeting vector, RAD51 (WT, KR, SP, KR/SP) or eGFP-RAD51 (WT and SP) with 5’ half of *miniHPRT* and 10 μg of pPGKcrepA were transfected to the cells and selected in 1x HAT.

#### Generation of mouse *RAD51*^*eGFP-RAD51(WT, SP)/-*^ ES cells

A guide RNA plasmid for mouse *RAD51* was generated by cloning guide sequences into pX330 (Addgene plasmid number 42230). The target sequences for gene editing were selected by using CRISPOR (www.crispor.tefor.net).^59^ The target sequences for the guide RNA in exon 4 were as follows: 5’-CAACTGAGTTTCACCAGCGC-3’. For mutating of the second copy of mouse *RAD51*, mouse *RAD51*^*+/eGFP-RAD51*^ ES cells were electroporated with the guide RNA (3 ug) that targets sequences in the fourth exon of mouse *RAD51* coupled with puro expression plasmid (2 ug). The gRNAs were: (forward) 5’-CACCCAACTGAGTTTCACCAGCGC-3’ and (reverse) 5’-AAACGCGCTGGTGAAACTCAGTTG-3’. The cells were seeded onto a 10-cm gelatin-coated plate. The next day, the cells were added and selected in 3 μg/mL of puromycin for 8 to 10 days. The resistant colonies were picked, expanded, and then screened by western blotting for deletion of mouse RAD51 expression (Figure S3B). The cells were lysed with NETN buffer (100 mM NaCl, 1 mM EDTA (pH 8.0), 20 mM Tris-HCl (pH 8.0), 0.5% NP-40 with 1x protease inhibitor cocktail) then sonicated, incubated on the ice for 30 min before centrifugation at 13,000 x g for 5 min at 4°C. Proteins were eluted in SDS sample buffer (60 mM Tris (pH 6.8), 2% SDS, 10% glycerol, 5% β-mercaptoethanol and 0.01% bromophenol blue) and boiled at 99°C for 10 min. 20 g of proteins were separated by 10% SDS-PAGE and examined by Western blotting with rabbit monoclonal Rad51 (D4B10, 1:1000), and mouse monoclonal β-actin (C4, 1:5000).

#### I-SceI based repair assays: DR-GFP, SA-GFP, EJ5-GFP

U2OS DR-GFP, SA-GFP and EJ5-GFP cells^56^ were a kind gift of Dr. Jeremy Stark (City of Hope, Duarte). To measure the repair of an I-SceI-generated DSB, U2OS cells carrying an integrated GFP recombination reporter were trypsinized and seeded 1.5x 10^5^ cells/well in 12-well plate. The next day, cells were transfected with 0.6 μg of the I-SceI expression vector (pCBASce) and 37.5 ng of each of hRad51 mutant expression vector (pCMV-hRAD51-WT and mutants) using 3.6 μg of Polyethyleneimine (Polyscience, Cat. #24765). After a 6-h incubation, the media was replaced with fresh media. Seventy-two hours after transfection, the cells were harvested and analyzed on a FACSVerse using BD FACSuite software (BD). The proportion of GFP-positive events (at least 50,000 singlet events were scored per sample) provided a measure of DSB repair.

Cell culture conditions: Human osteosarcoma cell line U2OS (ATCC HTB-96) grown in DMEM High Glucose w/o L-Glutamine w/o Sodium Pyruvate (Biotech, LM-D1108500) and 10% fetal bovine serum (Biotech, 10270-106), supplemented with 1X penicillin-streptomycine (Biotech, XC-A4122/100) and 1X L-Glutamine (Biotech, XC-T1715/100) at 37°C, 5% CO2 incubator.

To measure expression level of I-SceI and exogeneous RAD51, U2OS cells were seeded 3x 10^5^ cells/well in 6-well plate. Next day cells were co-transfected with the I-SceI expression vector (pCBASce) and 87.5 ng of the RAD51 expression vector. Seventy-two hours later cells were harvested and analyzed by Western blotting. Membranes were probed with antibodies against human RAD51 (Bio Academia 70-002), HA-tag to detect I-SceI (Abcam ab9110) and Actin as a loading control (Abcam ab184220).

#### Analysis of replication forks

DNA fiber analysis was used to measure RF restart/stalling and nascent strand degradation as described.^31^ Briefly, for replication fork dynamics, the cells were pulsed with 25 μM IdU for 20 min, treated with 0.5 mM HU for 1.5 hr and pulsed with 250 μM CldU for 20 min. To prepare DNA fiber spreads, the cells were dropped onto superfrost microscope slides (Thermo scientific) and dried for 6 min. Then, the cells were lysed with spreading buffer (200 mM Tris-HCl pH 7.4, 50 mM EDTA, 0.5% SDS) and incubated for 2 min. The slides were tilted at 15°, and the DNA fibers were spread slowly down to the end of slides. The fibers were fixed in methanol/acetic acid (3:1) at RT for 10 min and air-dried. The fibers were washed with ddH_2_O, rinsed with fresh 2.5 M HCl for 2 min, and denatured in fresh 2.5 M HCl for 1.25 hr. They were incubated in blocking buffer (1% BSA, 0.1% Tween 20 in PBS pH 7.4) for 0.5-1 hr and followed by incubation with rat α-BrdU (1:650) and mouse α-BrdU (1:650) in blocking buffer for 1 hr. The fibers were rinsed and fixed in 4% paraformaldehyde for 10 min. Then, they were incubated with secondary antibodies (anti-mouse AlexaFluor 488 and anti-rat Alexa Fluor 555, 1:500) for 1.5 hr and were mounted with Fluoroshield. The DNA fibers were captured with Axio Imager A2 at 63x magnification (Zeiss) and analyzed using Zen 2.3pro software (Zeiss). For nascent strand degradation, the cells were pulsed with 25 μM IdU for 30 min, pulsed with 250 μM CldU for 30 min. The cells were treated with 4 mM HU for 5 hr. The next procedures were followed as above. At least 1000 fibers and 129 fibers were counted and analyzed for replication fork dynamics and nascent strand degradation, respectively.

#### Immunoprecipitation of RAD51 variants

The indicated plasmids were co-transfected using Lipofectamine™ 3000 Transfection Reagent (invitrogen) according to the manufacturer’s instructions. After 6 hr, transfection reagents were removed and changed to fresh medium. After 48 h, HEK293T cells were harvested for further experiments. Cells were lysed with buffer X (100mM Tris-HCl (pH 8.5), 250 mM NaCl, 1 mM EDTA, 1% Nonidet P-40) and supplemented with the complete protease inhibitors cocktail (Roche) and PhosSTOP (Roche) for 1 hr at 4°C. Cell lysates were sonicated and centrifuged at 13000 rpm for 15 min at 4°C. Then cell lysates were incubated with Streptactin Sepharose® High Performance (GE Healthcare) for 1 hr at 4°C with constant rotation. After washing three times using buffer X, Streptactin-bound proteins were eluted by boiling samples in sample buffer (GenDEPOT), followed by immunoblotting with indicated antibodies.

The plasmids are as follows: pcDNA5/FRT/TO-Strep II human Rad51 (WT, S181P, K133R, KRSP), pCMV-3xFlag-EV (empty vector), human BRCA2 BRC3, and BRCA2 Ex27 (ALDFLSRLPLPPPVSPICTFVSPAAQKAFQPPRSCG).

#### RAD51 foci

The cells were grown onto a 15 mm coverslip (Chemglass Life Sciences) and treated with 1 μM CPT for 3 hr or 1 Gy X-ray (Faxitron CellRad) and 1 hr release. Then the cells were fixed with 4% formaldehyde in PBS for 20 min, rinsed with PBS and permeabilized with 0.5% Triton X-100 for 15 min. The cells were washed and blocked with 5% PBS/PBST for 30 min. Next, the cells were washed with PBST and incubated with anti-mouse AlexaFluor 488 (1:1000) for 45 min. After washing with PBST three times, the cells were stained with Vectashield mounting medium containing DAPI (Vector Laboratories). Images were captured by Axio Imager A2 at 63x magnification (Zeiss) and analyzed using Zen 2.3pro software (Zeiss). At least 184 cells were counted and the experiments were performed three times.

#### Metaphase spread (MPS)

This experiment has been described previously.^31^ Briefly, the cells were treated with 0.5 mM HU for 1.5 hr, 4 mM HU for 4 hr, 100 nM CPT for 16 hr, and 1 μM OLA for 16 hr and were incubated with 1 μg/mL of colcemid for 4 hr. The cells were harvested, resuspended with prewarmed 60 mM KCl and incubated at 37°C for 15 min. Then, the cells were collected in fresh fixative buffer (methanol/acetic acid 2:1) and were dropped onto Superfrost microscope slides to make metaphase spread. The slides were air-dried and aged in 100% methanol at RT overnight. The slides were incubated with 70% formamide in 2x SSC buffer (300 mM NaCl, 30 mM sodium citrate) at 72°C for 12 min. Then, the slides were incubated with 30% formamide, 0.27 μg/mL major satellite repeat probe (CY-3 5’-TGGAATATGGCGAGAAAACTGAAAATCATGGAA AATGAGA-3’) and telomeric probe (6-FAM 5’-(CCCTAA)7-3’) at 37°C for 25 min and mounted using Vectashield mounting medium containing DAPI (Vector Laboratories). The images were captured with Axio Imager A2 at 63x magnification (Zeiss), analyzed with Zen 2.3pro software (Zeiss) and ImageJ.^61^ At least 97 MPSs were scored and analyzed. The MPS survival fraction procedure was performed as described in the dose-response curve, but with minor modifications. On Day 0, 2x10^3^ cells were seeded onto a 24-well culture plate. On Day 1, cells were treated with HU (0.5 mM for 1.5 hr, 4 mM for 5 hr), 100 nM CPT for 16 hr, and 1 μM OLA for 16 hr. After treatment, the cells were washed with PBS twice and replenished with fresh media. On Day 6, the cells were harvested and counted with a hemacytometer.

#### Chromosome painting

Conditions are described in our previous publication.^31^

#### The *miniHPRT* LOF assay

The conditions for this experiment have been described previously.^2^ Briefly, the cells were kept in 1x HAT/1x HT for 5 days and were replenished with fresh media without HAT or HT. The next day, 2x10^5^ and 2x10^3^ cells were seeded onto a 10 cm culture plate with 10 μM TG and 6-well culture plate containing MEF feeder cells, respectively. After 7 days, the number of colonies were counted. The experiments were performed three times.

#### Protein expression and purification

Human RAD51 and its variants were expressed and purified as described previously.^29^ For Ex27-GST constructs, Ex27 domain of BRCA2 (ALDFLSRLPLPPPVSPICTFVSPAAQKAFQPPRSCG, this peptide is referred to as Ex27 in the text) was amplified from human genomic cDNA and cloned into pGEX-6P-1 vector. Ex27-GST, BRC4-GST and BRC3-GST constructs were then expressed and purified as previously described.^14^ A peptide corresponding to TR2 region of BRCA2 (aa 3265-3330) was custom-synthesized and used in experiments as indicated to supplement for truncated Ex27-GST protein.

#### *In vitro* pulldown assay

30 μg of BRC3-GST and Ex27-GST was incubated with 200 μl of glutathione agarose resin (Thermo Fisher) in T100 buffer (25 mM Tris-HCl, pH = 7.5, 100 mM KCl, 10 % glycerol, 0.5 % EDTA, 1 mM DTT, 0.01 % NP40) for 2 hr at 4°C with shaking. Beads were then washed twice with 1 mL of T100 buffer to eliminate unbound protein. Subsequently, 10 μL of beads coated with Ex27-GST or BRC3-GST were incubated with 2 μM RAD51 or its variants in buffer T100 in 20 μl final volume for 1 hr at 4°C. Following the incubation, supernatant was removed and beads were washed twice with 500 μL of T100 buffer. Beads were boiled in Laemmli buffer to obtain the bound fraction. Samples were loaded onto 12 % SDS-PAGE gel and stained with Coomassie Blue.

#### Electrophoretic mobility shift assay (EMSA)

RAD51 or its variants were diluted in T50 buffer (25 mM Tris-HCl, pH = 7.5, 50 mM KCl, 10 % glycerol, 0.5 % EDTA, 1 mM DTT, 0.01 % NP40) and incubated at 1.1 μM final concentration with 25 nM 5’FITC-labelled 90mer ssDNA [(5’FAM)AAATCAATCTAAAGTATATATGAGTAAACTTGGTCTGACAGTTACCAATGCTTAATCAGTGAGGCACCTATCTCAGCGATCTGTCTATTT] and 5.7 μM Ex27 peptide in D-loop buffer (10 mM Tris-HCl, pH = 7.5, 10 mM KCl, 1 mM MgCl_2_, 1 mM ATP, 1 mM DTT) for 10 min at 25°C. Following the incubation, increasing concentration of BRC3-GST protein trap was added and incubated for further 10 min at 25°C. Reactions were then resolved using 0.7 % agarose gels in 1xTAE at 4°C. Gels were scanned using FLA-9000 scanner and quantified using Multi Gauge V3.2 (Fujifilm).

#### D-loop formation assay

RAD51 or its variants were diluted in T50 buffer (25 mM Tris-HCl, pH = 7.5, 50 mM KCl, 10% glycerol, 0.5% EDTA, 1 mM DTT, 0.01% NP40) and incubated at 0.9 μM final concentration with 30 nM 5’FITC-labelled 90mer ssDNA [(5’FAM)AAATCAATCTAAAGTATATATGAGTAAACTTGGTCTGACAGTTACCAATGCTTAATCAGTGAGGCACCTATCTCAGCGATCTGTCTATTT] and increasing concentrations of Ex27 peptide (ALDFLSRLPLPPPVSPICTFVSPAAQKAFQPPRSCG, this peptide is referred to as Ex27 in the text) in D-loop buffer (10 mM Tris-HCl, pH = 7.5, 10 mM KCl, 2 mM MgCl_2_, 2 mM CaCl_2_, 2 mM ATP, 1 mM DTT) for 10 min at 37°C. 920 ng of pBluescript SK(–) (460 ng/μL) was then added and reaction was continued for another 15 min at 37°C. The samples were deproteinized with 0.1 % SDS and 10 μg proteinase K for 10 min at 37°C and resolved in 0.8 % agarose gels in 1X TAE. Gels were imaged on a FLA-9000 scanner (Fujifilm) and quantified with Multi Gauge V3.2 (Fujifilm).

#### ATPase assay

0.75 μM RAD51 or its variants were mixed with 7.5 μM Ex27 or BRC3-GST in A-buffer (50 mM Tris, pH 7.5, 50 mM KCl, 2 mM MgCl_2_) in the presence or absence of 120 nM (dT)63 homopolymer oligonucleotide. Mixtures were pre-incubated on ice for 15 minutes. Reactions were started by the addition of ATP up to 100 μM in the final volume followed by incubation at 37°C. Each time point, 50 μL of reaction was withdrawn and mixed with 12.5 μL of Goldmix/Accelerator (100:1) (Innova Biosciences PiColorLock Gold Colorimetric Assay kit) followed by 5 min incubation at 25°C. Then, 5 μL of Stabilizer was added for further 30 min. Standard curve was constructed according to manufacturer’s instructions. Fluorescence signal was measured using Infinity F500 microplate reader (Tecan Group Ltd.) in 96-well plates (25°C).

#### Stopped-flow rapid kinetics measurement

Stopped-flow experiments were performed similarly as described previously^62^ using an SFM-300 stopped-flow machine (Bio-Logic) fitted with a MOS-200 monochromator spectrometer (Bio-Logic) with excitation wavelength set at 545 nm. Fluorescence measurements were collected with a 550 nm long pass emission filter. All reactions were performed in Stopped Flow Buffer (50 mM Tris-HCl (pH 7.5), 10 mM MgCl_2_, 50 mM NaCl, 1 mM ATP) with 40 nM 5’-Cy3 fluorescently labelled (dT)79 homopolymer oligonucleotide and at 25°C. Proteins were added directly from concentrated stocks to concentration of 1.5 μM for RAD51 or its variants and 7.5 μM for Ex27 peptide. For all experiments, controls were also performed for buffer alone with and without DNA to confirm fluorescence signal stability over the time course of the experiments (data not shown). Fluorescence measurements for most experiments were collected according to the following protocol: (1) every 0.00005 s from 0-0.05 s; (2) every 0.0005 s from 0.05-0.56 s; (3) every 0.02 s from 0.56-60.54 s.

#### Bio-layer interferometry

All measurements were performed on a BLItz instrument (ForteBio) and in a buffer containing 50 mM Tris-HCl pH7.5, 50 mM KCl, 5 mM MgCl_2_, 2 mM ATP, and 0.05% Tween 20. For RAD51 filament formation, 15 nM of 3‘-biotinylated ssDNA (oligo-dT, 43-mer) was immobilized on streptavidin-coated sensors and incubated with 2 μM of RAD51 until equilibrium. The real-time kinetics of protein association and dissociation was measured as a change in optical thickness. The final concentration of BRCA2-Exon27 and GST-BRC4 used in experiments was 10 μM. The data were plotted and analyzed in GraphPad Prism.

### Quantification and statistical analysis

The statistical analysis was performed using Prism10 software (GraphPad). For dose-response curves, two-way ANOVA with Tukey’s multiple comparisons test was used. For DR-GFP, EJ5-GFP, and SA-GFP assays one-way ANOVA with Tukey’s multiple comparisons test was used. For RAD51 foci assay (Figure 4A), unpaired T-test was used. For DNA fiber assays, Chi-square with Yates’ correction and Fisher’s exact test (Figure 4B) and Kruskal-Wallis test with Dunn’s multiple comparisons (Figure 4C) were used. For the metaphase spread assay, Chi-square with Yates’ correction and Fisher’s exact test were used. For the *miniHPRT* loss of function assay, an unpaired t-test was used. Details regarding the number of replicates, corresponding p-values, and statistical tests are described in the figure legends.

## Supplemental information

Document S1. Figures S1‒S6

Table S1. Statistics for chromosomal abnormalities (related to Figure 5B) Table containing actual numbers of chromosomal abnormalities per MPS in tested conditions and related statistics. The sheet “each” lists types of abnormalities separately, while the sheet “all” shows data for all abnormalities combined. Chi-square test with Yates' correction was used to test statistical significance (Fisher’s exact test was used at indicated occasions when Chi-square test was not applicable due to too low expected frequency).
