# Supplementary material for: RAD51 separation of function mutation disables replication fork maintenance but preserves DSB repair
Source: iScience. 2024 Mar 16;27(4):109524. doi: 10.1016/j.isci.2024.109524 (PMC10993188; doi:10.1016/j.isci.2024.109524)
Supplement: Document S1. Figures S1‒S6 [file mmc1.pdf]

iScience, Volume *27*

Supplemental information

RAD51 separation of function mutation disables replication fork maintenance but preserves DSB repair

Mi Young Son, Ondrej Belan, Mario Spirek, Jakub Cibulka, Fedor Nikulenkov, You Young Kim, Sunyoung Hwang, Kyungjae Myung, Cristina Montagna, Tae Moon Kim, Lumir Krejci, and Paul Hasty

Supplemental Figures

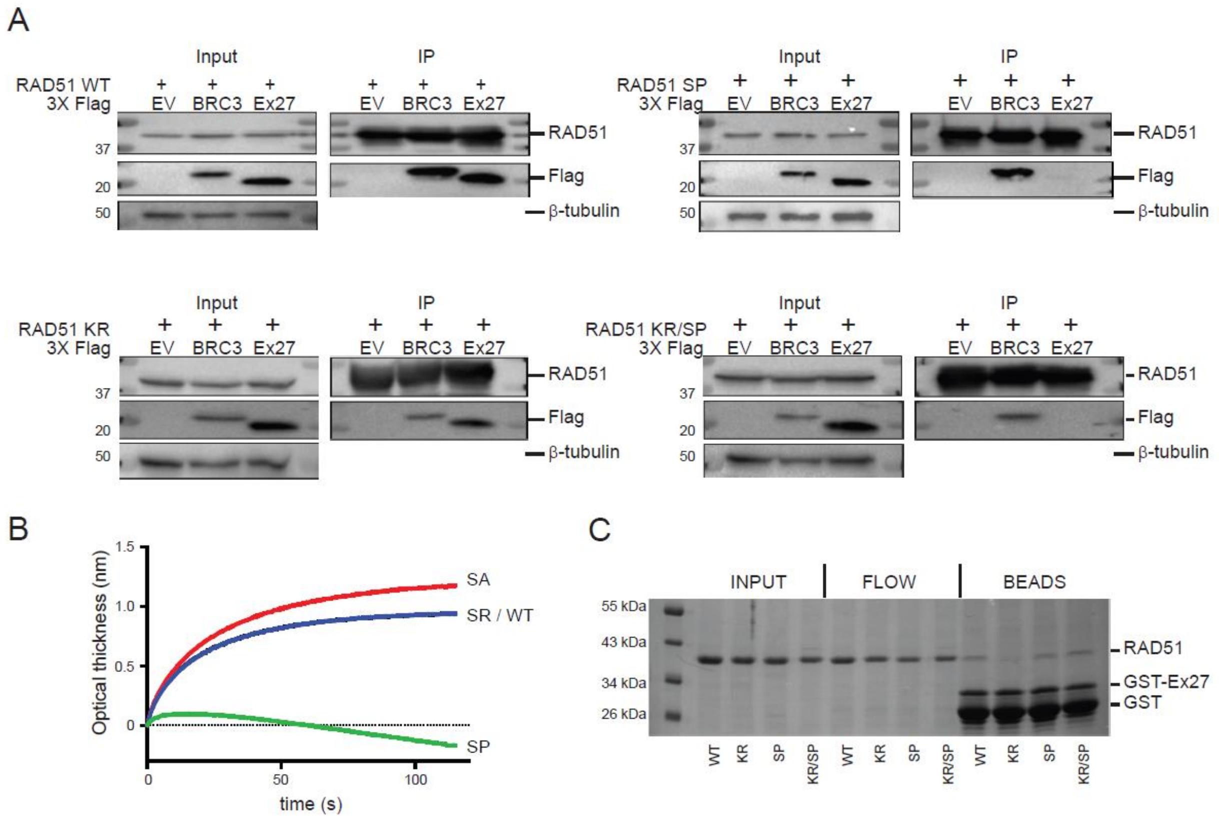

Figure S1 (Related to Figure 1). Association of RAD51 WT and its variants with Ex27. (**A**) Western blot analysis assessed the interaction of RAD51 WT and SP with FLAG-BRC3 and FLAG-Ex27. The results indicate that RAD51 WT immunoprecipitated with both FLAG-BRC3 and FLAG-Ex27. In contrast, SP is only immunoprecipitated with FLAG-BRC3 but fails to interact with FLAG-Ex27. EV - empty vector control. (**B**) Bio-layer interferometry was used to assess the binding of Ex27 to RAD51 (WT), S181P (SP), S181A (SA), and S181R (SR) filaments. (**C**) *In vitro* pulldown assay shows Ex27 binding to RAD51 WT, SA and SR, but not SP.

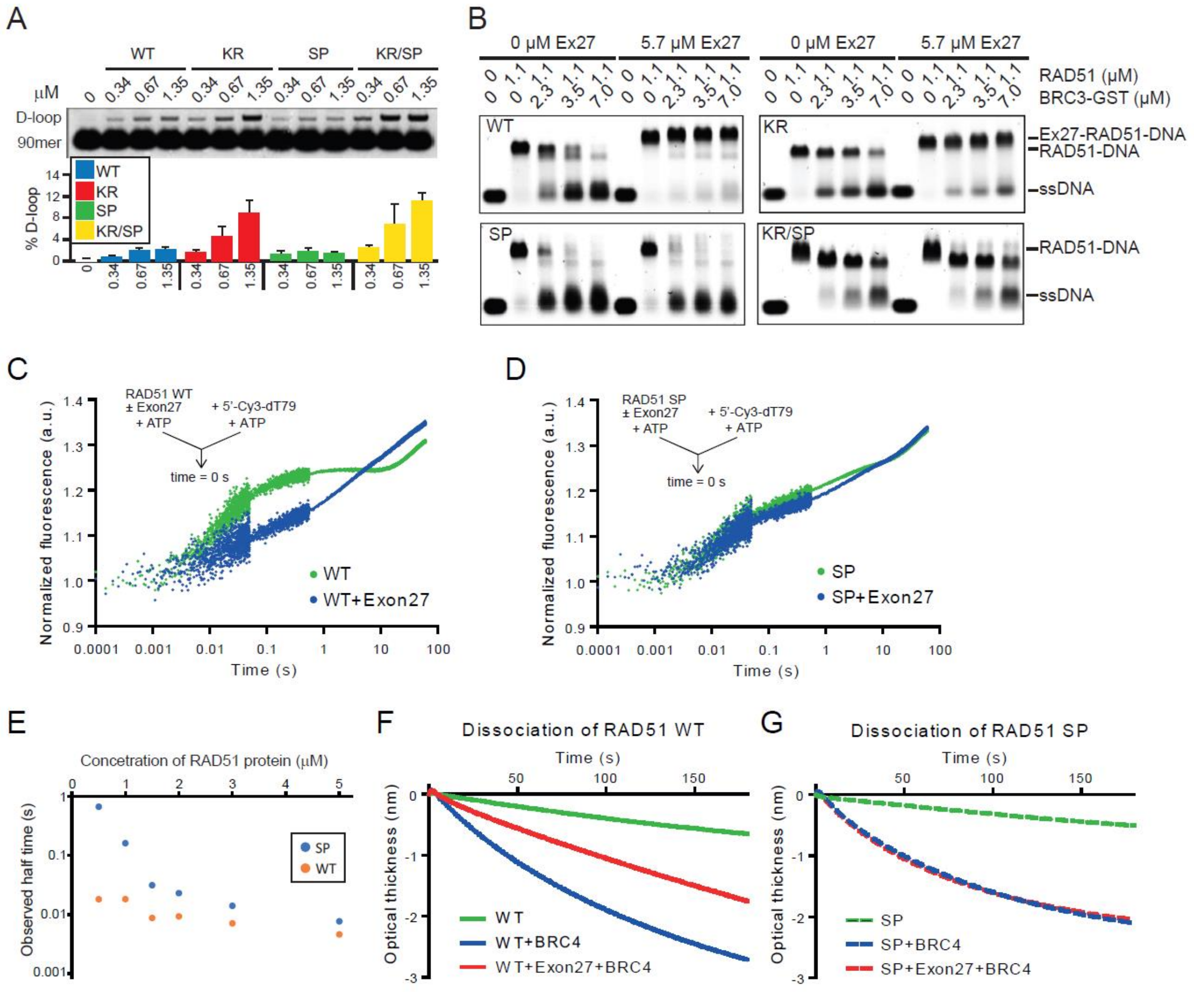

Figure S2 (Related to Figure 2). Characterization of RAD51 variants. (**A**) D-loop assay was performed in the presence of magnesium and ATP to compare the activities of RAD51 WT, SP, KR and KR/SP mutants. ssDNA and D-loop product are depicted. (**B**) Ex27 peptide (7.5 μM) failed to stabilize SP and KR/SP protein-DNA complexes from GST-BRC3-mediated disassembly. Representative agarose gel for Fig. 2D. (**C, D**) The stopped-flow analysis of RAD51 WT and SP proteins in the presence (blue) and absence (green) of Ex27. Compared to WT (**C**), SP (**D**) showed an increased overall binding amplitude and decreased binding rate, which was not affected by Ex27. (**E**) Analysis of the half times of RAD51 WT and SP mutant filament assembly from C and D. (**F, G**) RAD51 WT (**F**) and SP (**G**) filament dissociation monitored by BLI. The dissociation of RAD51 from ssDNA was induced by the addition of GST-BRC4 peptide in the presence (red) or absence (blue) of Ex27. A decrease in optical thickness

was monitored over time. A control curve (green) depicts corresponding RAD51 dissociation without GST-BRC4.

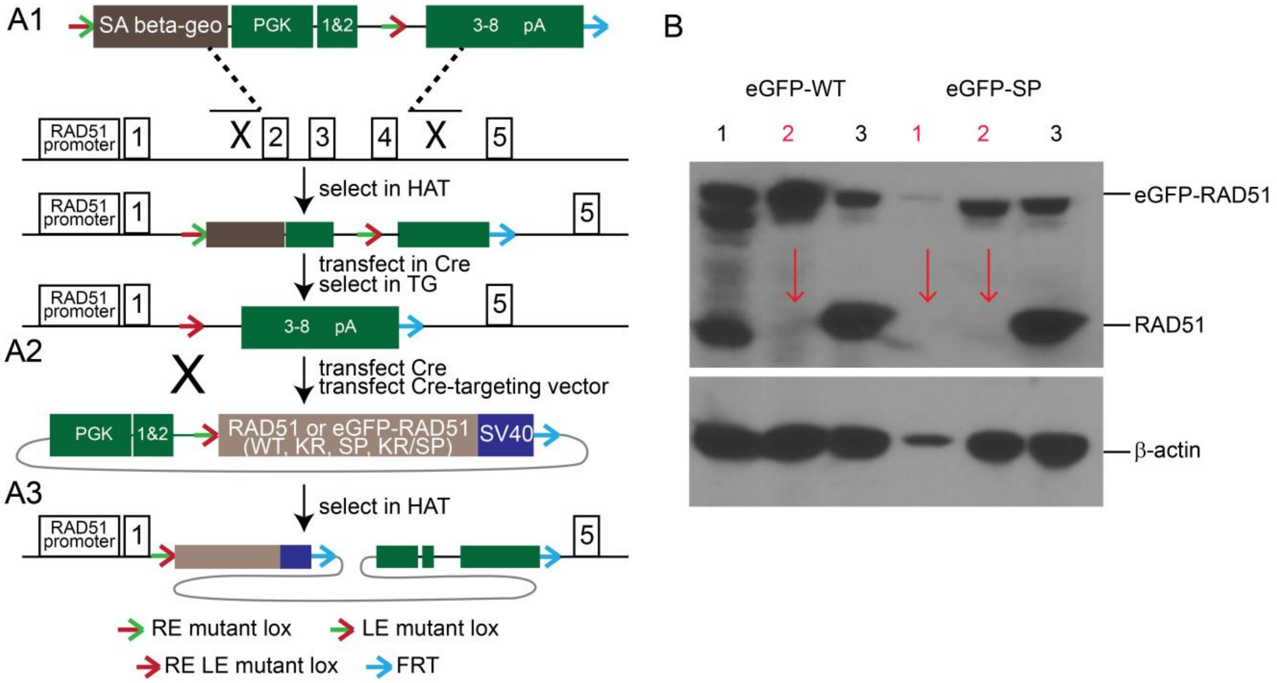

Figure S3 (Related to Figure 3). The strategy of knockout/knockin into *RAD51* locus. (**A1**) Knockout of one copy of mouse *RAD51* [1]. Replacement of *RAD51* exons 2-4 with *SAβgeo-miniHPRT*. *SAβgeo* is a fusion of *β galactosidase* and neomycin phosphotransferase with a splice acceptor (SA) instead of a promoter. Cells will survive G418 selection only if a promoter/splice donor is trapped [2]. *MiniHPRT* contains a phosphoglycerate kinase (PGK) promoter with an intron that separates exons 1&2 (small box) from exons 3-8 and polyadenylation sequences (large box) [3,4]. An *RE mutant loxP* (red/green arrow) [5] flanks the 5' end and a second *RE mutant lox* is located in the intron of *miniHPRT*. An FRT (blue arrow) flanks the 3' end. Selection is performed in HAT (hypoxanthine, aminopterin, thymidine) and is followed by a screen for targeted clones with PCR. (**A2**) Removal of the 5' half of *miniHPRT*. After transfection with Cre-recombinase, the selection is performed in 6-thioguanine (TG). Screen for removal of 5' *miniHPRT* is performed by PCR. (**A3**) Knockin of Cre-mediated targeting vector that contains RAD51 (WT, KR, SP, KR/SP) or eGFP-RAD51 (WT, SP). After selection in HAT, a screen for knockin is performed by PCR. (**B**) Knockout of wildtype copy of mouse *RAD51* in cells expressing either eGFP-WT or eGFP-SP. Western blot using RAD51 antibody (RAD51) showing deletion of wildtype mouse RAD51 in eGFP-WT (lane 2) and eGFP-SP (lanes 2 & 3) cells. Arrows are pointing to the location where the RAD51 band should be seen.

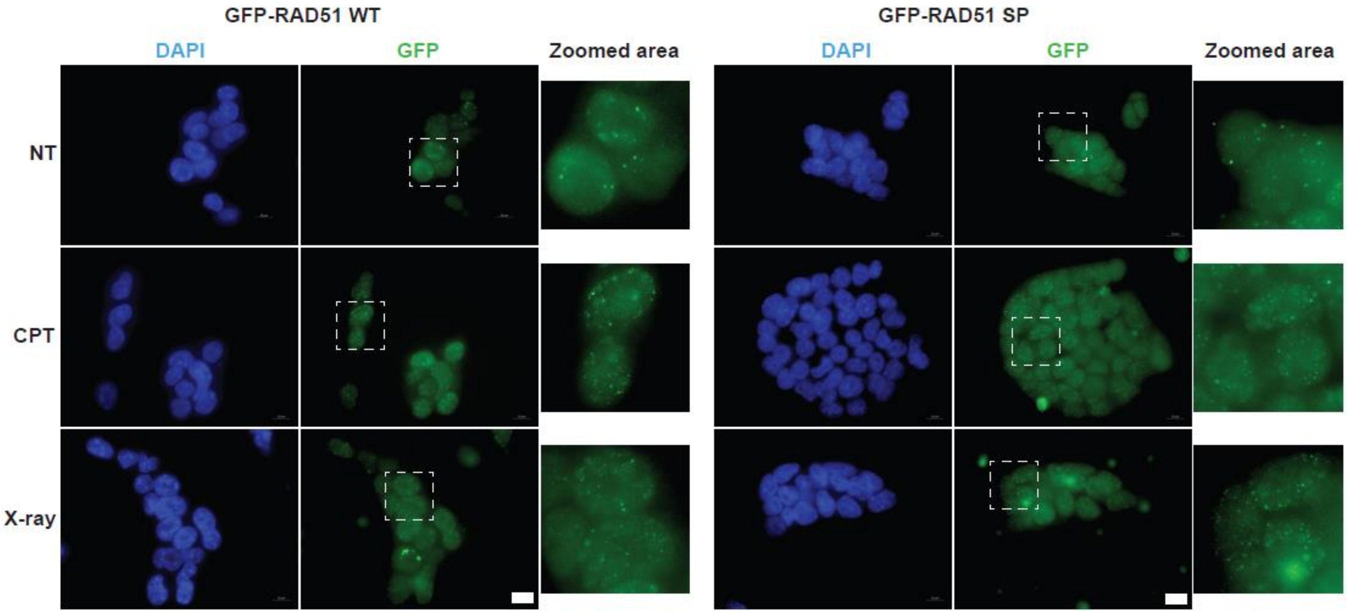

Figure S4 (Related to Figure 4). RAD51 foci analysis. Representative pictures for the graph presented in Fig 4A. White bar represents 10 μm. RAD51 (green), and DAPI (blue) images were assessed.

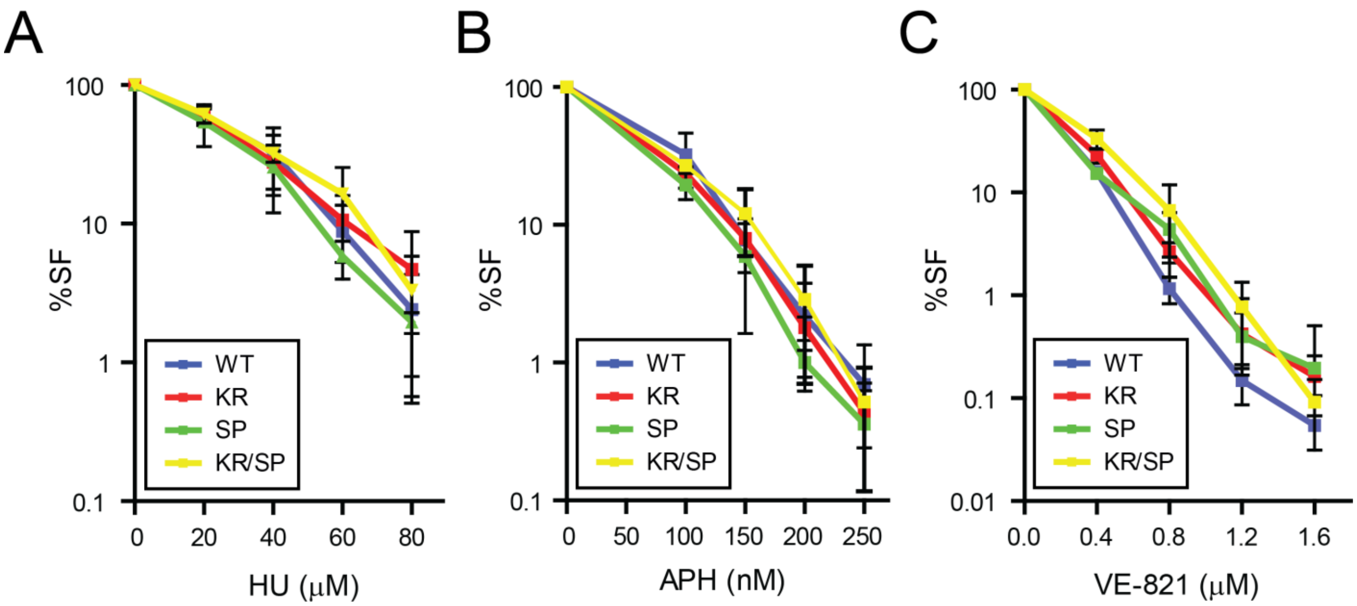

Figure S5 (Related to Figure 4). Sensitivity of RAD51 mutants to replication poison. (**A-C**) Dose response of RAD51 mutant cells exposed for 5 days to hydroxyurea (HU, 20-80 μM) (**A**), aphidicolin (APH, 100-250 nM) (**B**) and VE-821 (0.4-1.6 μM) (**C**). Survival fraction, SF. Mean ± SD, n=3, Statistics: Unpaired T test.

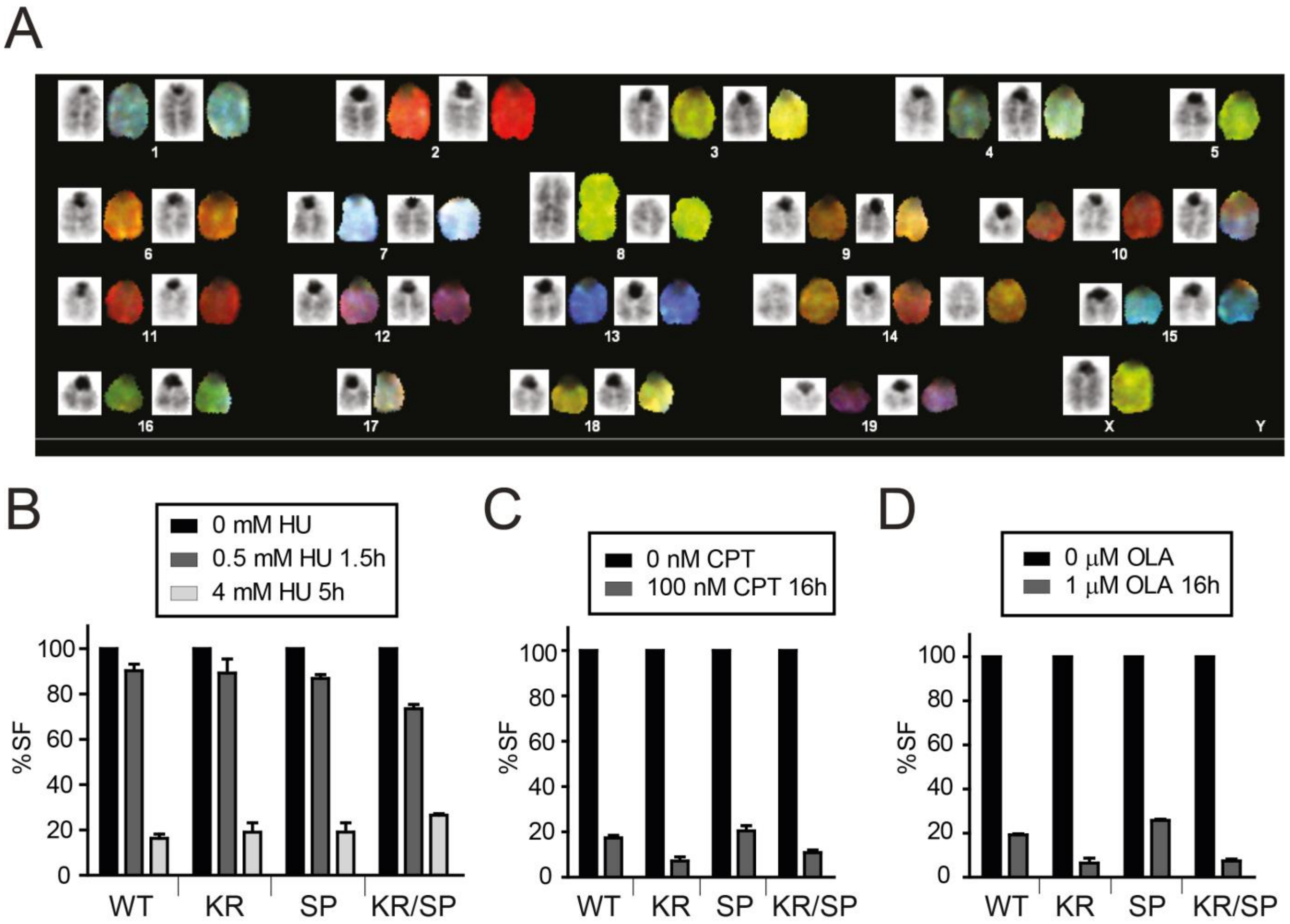

Figure S6 (Related to Figure 5). Metaphase spread analysis. (**A**) Chromosome painting analysis on RAD51 SP cells showing an RbT on chromosome 8. (**B-D**) Graphs showing the percentage of survival fractions (SF) for the metaphase spread analysis for cells exposed to HU (**B**), CPT (**C**) and OLA (**D**). Mean ± SD, n=3.

Supplemental references

1. Kim, T.M., Ko, J.H., Hu, L., Kim, S.A., Bishop, A.J., Vijg, J., Montagna, C., and Hasty, P. (2012). RAD51 mutants cause replication defects and chromosomal instability. Mol Cell Biol *32*, 3663-3680. 10.1128/MCB.00406-12.
2. Friedrich, G., and Soriano, P. (1991). Promoter traps in embryonic stem cells: a genetic screen to identify and mutate developmental genes in mice. Genes Dev *5*, 1513-1523.
3. Reid, L.H., Gregg, R.G., Smithies, O., and Koller, B.H. (1990). Regulatory elements in the introns of the human HPRT gene are necessary for its expression in embryonic stem cells. Proc Natl Acad Sci U S A *87*, 4299-4303.
4. Holcomb, V.B., Kim, T.M., Dumitrache, L.C., Ma, S.M., Chen, M.J., and Hasty, P. (2007). HPRT minigene generates chimeric transcripts as a by-product of gene targeting. Genesis *45*, 275-281.
5. Araki, K., Araki, M., and Yamamura, K. (1997). Targeted integration of DNA using mutant lox sites in embryonic stem cells. Nucleic Acids Res *25*, 868-872.
